# Clowns Benefit Children Hospitalized for Respiratory Pathologies

**DOI:** 10.1093/ecam/neq064

**Published:** 2011-03-15

**Authors:** Mario Bertini, Elena Isola, Giuseppe Paolone, Giuseppe Curcio

**Affiliations:** ^1^Department of Psychology, “Sapienza” Università di Roma, Via dei Marsi 78, Italy; ^2^Department of Paediatrics, Ospedale San Camillo, Piazza Forlanini 1, 00151 Rome, Italy; ^3^Dipartimento di Scienze della Salute, Università dell'Aquila, Via Vetoio (Coppito 2), 67010 Coppito, AQ, Italy; ^4^Department of Health Sciences, Università degli Studi dell'Aquila, Via Vetoio (Coppito 2), L'Aquila, Italy

## Abstract

The study aims at evaluating health-generating function of humor therapy in a hospital ward hosting children suffering from respiratory pathologies. The main scope of this study is to investigate possible positive effects of the presence of a clown on both the clinical evolution of the on-going disease, and on some physiological and pain parameters. Forty-three children with respiratory pathologies participated in the study: 21 of them belonged to the experimental group (EG) and 22 children to the control group (CG). During their hospitalization, the children of the EG interacted with two clowns who were experienced in the field of pediatric intervention. All participants were evaluated with respect to clinical progress and to a series of physiological and pain measures both before and after the clown interaction. When compared with the CG, EG children showed an earlier disappearance of the pathological symptoms. Moreover, the interaction of the clown with the children led to a statistically significant lowering of diastolic blood pressure, respiratory frequency and temperature in the EG as compared with the control group. The other two parameters of systolic pressure and heart frequency yielded results in the same direction, without reaching statistical significance. A similar health-inducing effect of clown presence was observed on pain parameters, both by self evaluation and assessment by nurses. Taken together, our data indicate that the presence of clowns in the ward has a possible health-inducing effect. Thus, humor can be seen as an easy-to-use, inexpensive and natural therapeutic modality to be used within different therapeutic settings.

## 1. Introduction

Psychobiological research, ranging from Selye's classical studies on stress [1] to the more recent ones in the field of psycho-neuro-endocrinology and psycho-immunology has thoroughly described the impact of “negative emotions", such as fear, anger/rage and anxiety on animals' and human health. Much less attention, however, has been dedicated to the way positive emotions can contribute to producing beneficial effects for health enhancement [2]. The last few years have seen a flourishing number of studies on humor considered as a valid moderator of stressful or painful situations. Some findings support the benefits of humor and laughter in areas of cardiac rehabilitation [3], pain perception [4, 5], discomfort thresholds [6], coping with stress [7], stress hormone modulation [8, 9] and immune enhancement in children and adults [10–13]. Furthermore, recently several exhaustive reviews have been proposed [12–17], also highlighting critical aspects of this research field (see e.g., [18]).

Based on these premises, more practical initiatives aimed at using humor in its various forms to stimulate patients' vitality and recovery, have been cropping up. A recent study [19] describes the effects of a 6-week study with one clown visit per week in a ward for acutely ill geriatric patients. The results showed more positive attitudes in patients after the experimental manipulation. An ever growing interest in the presence of clowns in hospital wards, and in particular in pediatric departments, appears evident. The existing literature on humor as a tool of coping with stressful situations provoked by hospitalization highlights certain benefits, both in somatic and psychic terms. A study of the effects of clown's presence on children's preoperative anxiety during the induction of anesthesia showed that the “clown group" was significantly less anxious compared with a control group [20]. Likewise, humor interventions have shown a decrease in children's anxiety and an increase in their cooperation in a variety of procedures [21] such as indwelling catheter access [22] and bone marrow transplantation [23]. The study of effects of humor on various health-related outcomes in healthy populations is in its early stages, and there is still no real research documenting the benefits in a clinical population, such as in oncology patients [22]. A recent review also affirmed that empirical studies are still lacking, and that published works show serious methodological shortcomings [18].

The present study aims to observe the impact of humor induced by the presence of clowns on the health of children during hospitalization in a pediatrics respiratory department, with two specific objectives: (i) assessing the benefit of the clown intervention on the patients' clinical progress; (ii) examining the possible changes directly brought about by the clown interaction on some physiological and pain parameters of the child, in order to contribute to understanding the hypothesized beneficial effects on health.

## 2. Methods

### 2.1. Participants

Before admission to the hospital, on the basis of the waiting list, each eligible patient was labeled with a number code (1, 2,…, 44) and by means of a custom computerized software, randomly assigned to one of the two groups (experimental or control), controlling for an adequate matching with respect to participants' age and family's socio-economic and cultural background. On the basis of this random grouping, the patients were admitted to the hospital in the two separate periods of the study (see below). When hospitalized, all the 44 children agreed to participate in the study and an informed consent was signed by both their parents. Due to data loss of one patient, 21 instead of 22 children (12 boys and 9 girls; mean age in years = 7.71 ± 2.47) belonged to the experimental group (EG), and 22 (13 boys and 9 girls; mean age in years = 7.54 ± 2.06) belonged to the control group (CG). The identified pathologies reveal a substantially equal distribution in both groups. In the EG, 10 children were suffering from pharyngitis, tonsillitis, tracheitis and laryngo-tracheitis, and 11 children were suffering from pneumonia, bronchitis and broncho-pneumonia. In the CG, 10 children were suffering from the former pathologies and 12 from the latter. The procedure was approved by the local Ethics Committee.

### 2.2. Clown Interaction Program

The research was carried out in the Paediatrics Department of “San Camillo" Hospital in Rome during the month of October 2008: the first half of the month was dedicated to the CG while the second to the EG. The 21 EG children during their hospitalization interacted with two clowns experienced in the field of pediatric intervention, while the 22 CG children did not have any contact with the clowns. Each session was planned for seven to eight children at a time. The CG and EG children were studied in the same ward.

The clowns' intervention in the hospital ward consisted of sessions lasting almost 3 h (from 2 p.m. to 5 p.m.) performing various activities, and adapting the play to each patient's age and psychological condition as much as possible. The clowns arranged common play sessions with patients who were free to move in their ward, and used various methods for entertaining the child as magic tricks, gags, puppets, soap bubbles, games, word games. At first, the children assisted the clown and then were invited to play with the clowns, repeating all the games they liked. The children's parents were not present in the playing rooms. Although the clowns' presence in the ward was relatively limited, in the other days there were traces of their presence in each hospital room (e.g., drawings, balloons) and the hospital environment appeared different. All the children of the EG met the clowns only once because of their short stay in the hospital.

### 2.3. Clinical Assessment

Assessment of the clinical progress was carried out by analyzing the following parameters: (i) duration of stay in the hospital (number of days), (ii) duration of the fever period (number of days), (iii) time taken to achieve clinical recovery (number of days), as measured by the length of time needed for the symptoms (e.g., wheezing, bronchus) to disappear and by analyzing the patient's chest report. When the audio and chest reports were normalized, the data were written down in the patients' clinical reports. The assessment of the patients' clinical progress, deduced from the daily reading of clinical records, was carried out by a person not involved in the study.

### 2.4. Physiological Assessment

To evaluate the direct impact of the clowns' activities on the children, a series of physiological measurement were recorded before the play sessions (at around 01.30 p.m.) and half-an-hour after the interaction (at 05.30 p.m.). All the measurements were carried out on both experimental and control groups in a comparable time window. Moreover, as a control, these same evaluations were carried out also during the days before and after the experimental day (i.e., when the clowns were not present in the ward). Several measurements were collected: (i) systolic and diastolic blood pressure (mmHg); (ii) heart rate (number of beats per minute); (iii) respiratory frequency (number of breaths per minute); and (iv) axillary temperature (°C).

### 2.5. Pain Evaluation

Following the same schedule and procedure of physiological recordings (see above), pain was also evaluated. Usually, with young children both subjective (self rated) and nurses rated scales are used by asking to rate the current state on a continuum from “no hurt" (no pain at all) to “hurts worst" (very painful). The following three tests were used, before and after the play sessions:

1. The Wong/Baker pain rating scale [24, 25], presents a series of six faces with varying expressions ranging from no pain (“no hurt", score 0) to a lot of pain (“hurts worst", score 10). More specifically, face 0 shows a smiling face indicating no pain at all, face 2 a non-smiling face indicating a little pain, face 4 a little more pain, face 6 even more pain, face 8 a lot of pain, face 10 is contorted in pain indicating as much pain as one can imagine. The participants were asked to choose the face that best described how much pain they felt. We used this scale as a visual measure of individual pain.
2. A pain self-evaluation numeric scale (NRS-11; [26]), NRS-11 is a 11-point numeric rating scale ranging from 0 (no pain) to 10 (worst possible pain): a score of 0 represents no pain, 1–3 mild pain, 4–7 moderate pain and ≥8 severe pain. In this case, each child was asked to indicate the number that best described his/her current level of pain. We used this scale as a numerical measure of individual pain assessment.
3. The CHEOPS scale (Children's Hospital of Eastern Ontario Pain Scale; [27]), is a behavioral scale originally developed for evaluating postoperative pain in young children. It can be used to monitor the effectiveness of interventions for reducing the pain and discomfort. It is an scale containing six behavioral subscales (Cry, Facial, Child Verbal, Torso, Touch, Legs) and some possible indicators: for example the subscale Facial can be defined as “smiling" (score: 0), “composed" (score: 1) or “grimace" (score: 2). The CHEOPS pain score is the sum of points for all six subscales; the minimum score is 4 while the maximum is 13. The data was collected by the division nurses.

### 2.6. Data Analysis

The data for each clinical assessment variable (Days of hospitalization, Fever and Clinical improvement) were submitted to a one-way analysis of variance (ANOVA) comparing the CG and EG. Moreover, for each psychophysiological (systolic and diastolic blood pressure; heart rate; respiratory frequency; axillary temperature) and psychometric variable (Wong/Baker scale; Cheops scale, Pain self-evaluation numeric scale) a mixed-model ANOVA Group (EG, CG) × Time (Pre-, Post-clown intervention) was carried out separately for the day of clown intervention and the day before and after. Statistical analyses were carried out using Statistica 6.1 software (StatSoft) for Windows.

## 3. Results

### 3.1. Clinical Improvement due to Clown Interaction

**Figure 1:**
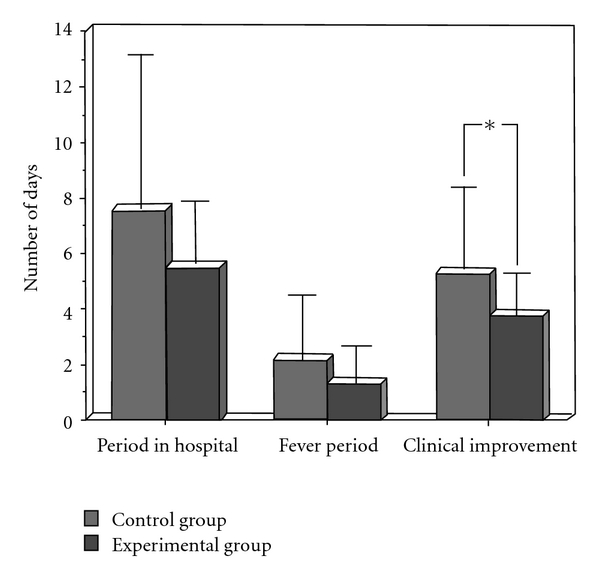
Mean and SD of clinical assessment measures (duration of stay in the hospital, duration of the fever period, clinical improvement—that is, time needed for healing) in the CG and EG (*P < .03).

The one-way ANOVA carried out on the days of hospitalization variable showed no significant main effect (*F*_1,41_ = 2.42; *P* = n.s.), although the mean duration of the hospitalization period for the EG was shorter (mean ± SD = 5.52 ± 2.25 days) than for the CG (7.55 ± 5.52 days) as reported in Figure 1. The same hypothesized trend was observed with the Fever duration variable (*F*_1,41_ = 2.30; *P* = n.s.; EG = 1.33 ± 1.27; CG = 2.18 ± 2.24 days, see Figure 1). A statistically significant effect (*F*_1,41_ = 4.98; *P* = .03) was observed for the Clinical improvement with a shorter time needed for healing in the EG (3.72 ± 1.45 days) than in the control group (CG = 5.36 ± 2.97 days), as shown in Figure 1. 

### 3.2. Improvement of Autonomic and Temperature Parameters after Clown Interaction

**Figure 2:**
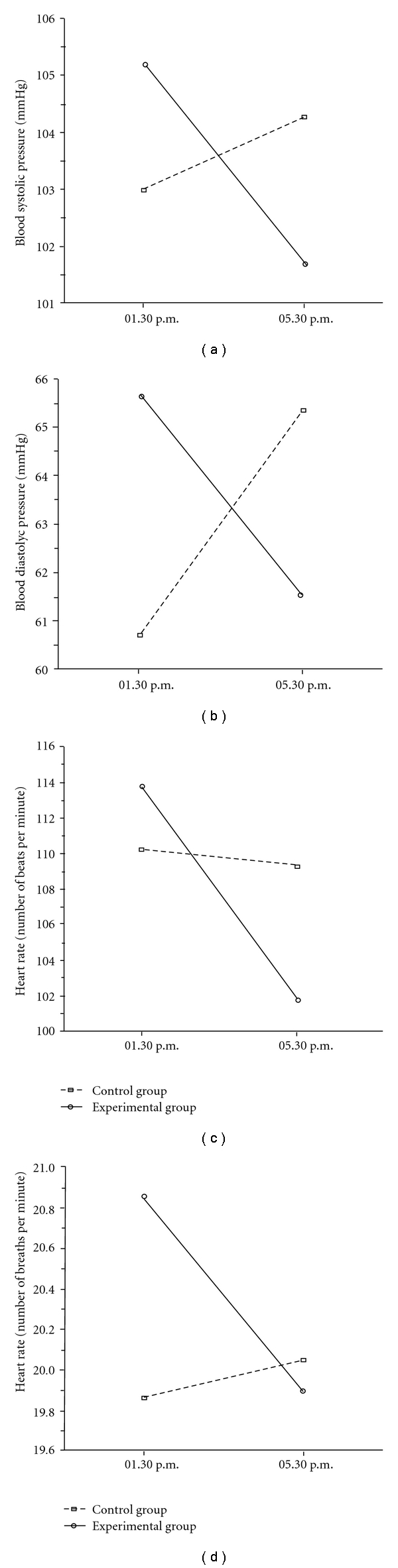
Results of Group × Time interactions highlighted by the ANOVAs carried out on physiological variables on the day of clown interaction. (a) The difference between the CG and EG for blood systolic pressure, recorded before and after the experimental intervention; (b) the statistically significant effect (*P* < .02) for blood diastolic pressure recorded before and after the experimental intervention in the CG and EG; (c) heart rate, recorded before and after the experimental intervention; (d) a statistically significant interaction (*P* < .002) in the respiratory frequency recorded before and after clown intervention in the CG and EG.

The mixed-model ANOVA carried out on systolic blood pressure, showed no significant main or interaction effects, either for the day of clown intervention or for the days before and after it. Nonetheless, the mean values of systolic blood pressure showed a sharp decrease in the EG with respect to the CG, only on the day of clown intervention (Figure 2(a)). 

The same analysis carried out on diastolic blood pressure, showed a significant Group × Time interaction (*F*_1,41_ = 6.10; *P* = .017) on the day of clown intervention, indicating that the diastolic blood pressure significantly increased in the CG (*post-hoc* LSD test *P* < .005) and decreased in the EG (*post-hoc* LSD test *P* < .003; Figure 2(b)). No other significant effects or interactions were observed in the day before and after the clown intervention.

With respect to heart rate, no significant main or interaction effects were found. Nonetheless, here too, solely with regard to the day of clown interaction the mean number of beats per minute showed a sharp but not significant decrease in the EG with respect to the CG (Figure 2(c)).

The mixed-model ANOVA on respiratory frequency, showed a significant main effect for Time (*F*_1,41_ = 4.82; *P* = .034), indicating a decreasing trend of the number of breaths per minute (Pre = 20.36 ± 2.14; Post = 19.97 ± 1.86). Moreover, a statistically significant Group × Time interaction (*F*_1,4_ = 10.45; *P* = .002) was also found, indicating an increasing respiratory frequency in the children of the CG and a decrease in the EG (Figure 2(d)). Also in this case, no other significant effects or interactions were observed on either the day before or after the clown intervention.

**Figure 3:**
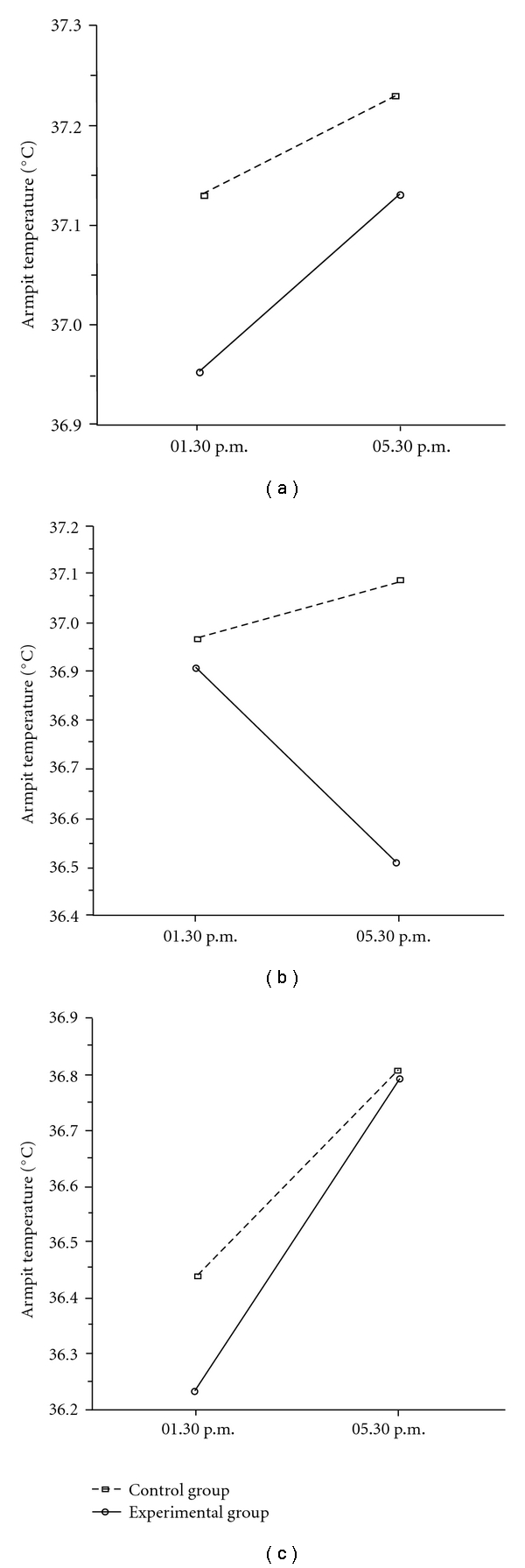
Results of Group × Time interactions highlighted by the ANOVAs carried out on axillary temperature. (a) The daily trend of temperature in the CG and EG recorded the day before the experimental clown intervention; (b) the significant (*P* < .008) difference of axillary temperature between the CG and EG recorded the day of clown intervention as well as before and after the clown's presence in the ward; (c) the temperature in both the CG and EG, recorded the day after clown intervention.

Finally, the analysis carried on temperature measured from the armpit, showed a very interesting scenario, especially if we look at the data trend during the 3 days, from 1 day before to 1 day after the day of clown interaction. The day before the clown intervention, a trend toward an increase in axillary temperature was observed in the EG and CG (Figure 3(a)). This trend (although statistically not significant) was largely expected due to the afternoon physiological rise in temperature observed daily. On the day of clown interaction, the Group × Time interaction result was statistically significant (*F*_1,41_ = 7.67; *P* = .008), with a marked decrease of temperature limited to the EG (*post-hoc* LSD test *P* < .001; Figure 3(b)). Finally, on the day after the clown intervention, axillary temperature again tended to increase in the late afternoon in both the EG and CG (Figure 3(c)) as observed in the absence of clown intervention. 

### 3.3. Reduction of Pain as a Function of Clown Interaction

**Figure 4:**
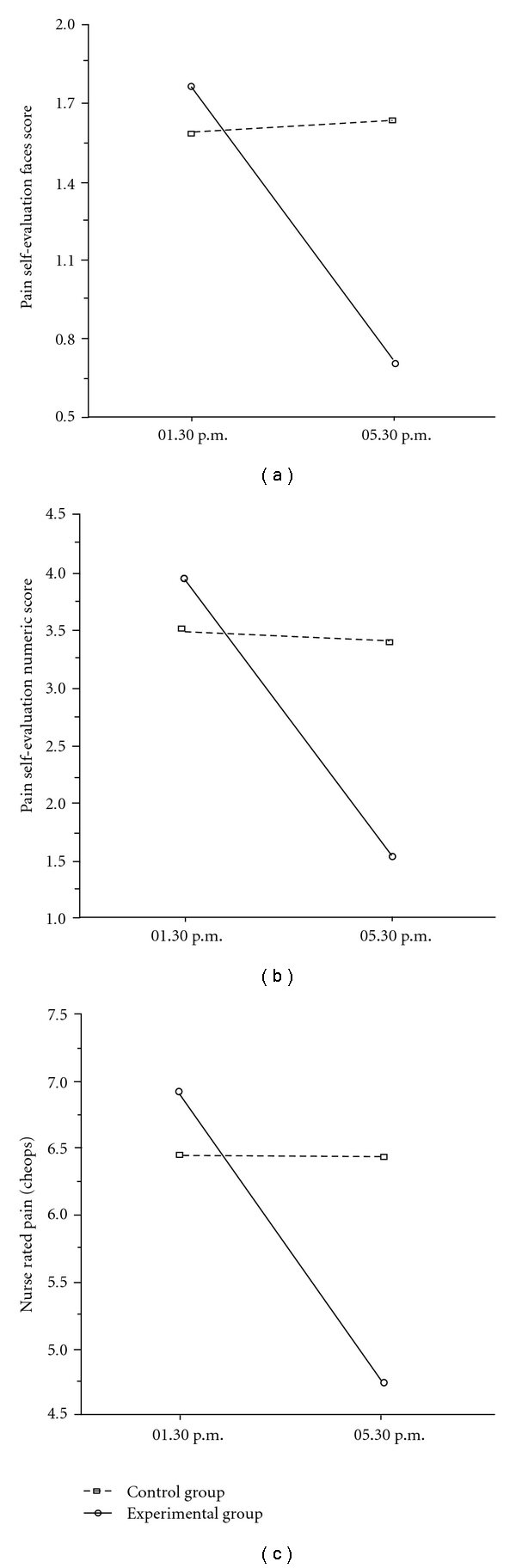
Results of statistically significant Group × Time interactions highlighted by the ANOVAs carried out on pain scales. (a) The difference between the CG and EG for the Wong/Baker faces pain scale, evaluated before and after the experimental clown intervention; (b) the difference between the CG and EG for pain self-evaluation numeric scale, evaluated before and after the experimental clown intervention; (c) the difference between the CG and EG for the Cheops evaluation of pain, evaluated before and after the experimental clown intervention.

The ANOVA carried out on the Wong/Baker faces scale scores, showed a significant main effect for Time (*F*_1,41_ = 18.51; *P* = .0001), indicating a decreasing trend of pain sensation from Pre- (1.68 ± 1.19) to Post-clown intervention (1.17 ± 1.02). Also the Group × Time interaction resulted statistically significant (*F*_1,41_ = 22.01; *P* = .00003), with a strong decrease of scores in the EG as a function of treatment (Figure 4(a)) and a slight increase in the CG. 

A similar scenario was seen with the Pain self-evaluation numerical scale. A significant main effect for Time (*F*_1,41_ = 57.54; *P* < .00001), indicated a trend of decreasing self evaluated pain from Pre- (3.75 ± 2.38) to Post-treatment (2.49 ± 2.26). Moreover, a significant Group × Time interaction (*F*_1,41_ = 49.39; *P* < .00001), showed a strong decrease of self-evaluated pain in the EG, and a slight decrease in the CG (Figure 4(b)).

Finally, these results were confirmed by the nurse rated pain levels (Cheops). A significant main effect for Time (*F*_1,41_ = 29.5; *P* < .00001) was observed, indicating a trend of decreasing pain evaluation from Pre- (6.68 ± 2.27) to Post-treatment (5.61 ± 5.63). The statistically significant Group × Time interaction (*F*_1,41_ = 29.49; *P* < .00001), showed a strong decrease of nurse rated pain in the EG, while the CG remained unchanged (Figure 4(c)).

## 4. Discussion

It is surprising to note that the subject of biological correlates of emotions such as joy, sense of humor, contentment and interest, and their positive influence on health have been neglected in research. Only very recently humor has come under the purview of neuroscience [28]. The growing movement from the so called “disease model" toward the “health model" [29–31] explains the increasing interest in the positive dimensions of health and their possible healing properties in the hospital setting. One particular question is the widespread use of clowns in pediatric wards despite the shortage of studies showing the objective utility of their intervention.

To provide empirical evidence in this area, the first aim of this study was to show whether a clown intervention in a pediatric respiratory pathology ward could facilitate the healing process of the juvenile patients. An overall analysis of the results tends to support this hypothesis. When compared with a CG, the clowns' interaction with the group of children suffering from respiratory pathologies, led to an earlier disappearance of the pathological symptoms.

The second aim was to investigate the possible changes induced by the clown intervention on some physiological and pain parameters of the child. Compared with the CG, we expected the children of the EG to present a higher level of general relaxation (or a lower level of stress). In line with the hypothesis, we found a statistically significant lowering of diastolic blood pressure, respiratory frequency, and temperature in the EG as compared to the CG. The other two parameters of systolic pressure and heart frequency also had showed similar trends but did not reach statistical significance. Collectively, the decrease in these physiological parameters suggests a general reduction of stress levels. Of particular interest is the decrease in the children's temperature measured in the EG during their interaction with clowns, observed during a critical time window (1.30–5.30 p.m.) when a general increase in temperature was expected. The robustness of this finding is intensified by the observation that the same expected increase in temperature measured in the CG, was also found in the EG on both the day before and after the clown interaction. It is hard to put forward any sound explanation for this particular phenomenon: it is, however, an empirical finding which certainly deserves closer examination. To our knowledge, in fact, it is the first time that an association between humor and fever reduction has been reported in the literature. The data reporting pain perception both self- and nurse rated, also support a general decrease in distress level. The children exposed to clowns' interaction reported less somatic pain with respect to those of the CG. The same reduction in pain was indicated by the nurses' evaluation conducted by means of the Cheops scale.

Altogether, the physiological and psychological observations appear to show that the children's bodies enjoyed a psychophysiological positive state during the clowns' presence, and would thus seem to agree with the opinion of some authors that humor can lead to muscle relaxation, with a corresponding decrease in heart rate, respiratory rate and blood pressure [12, 13, 16]. As matter of fact, all the patterns of Figure 2 appear to be a good example of a well-functioning eustress response.

**Figure 5:**
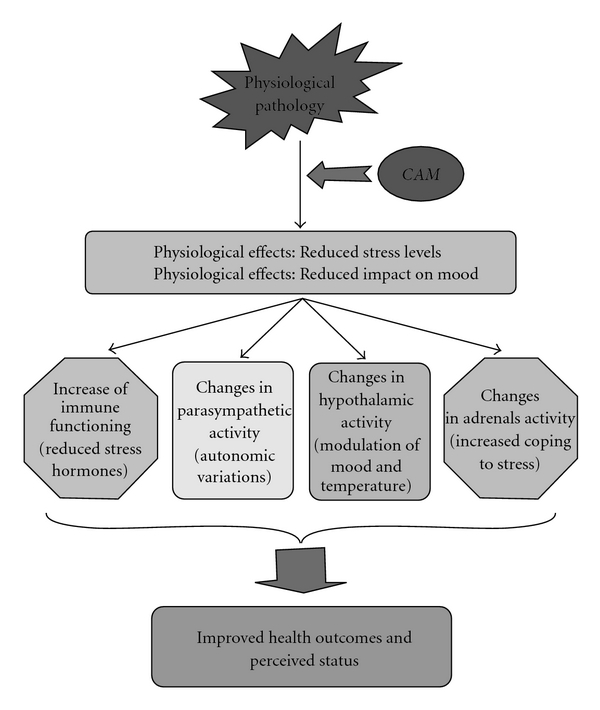
Example of some potential mechanisms by which humor can contribute to physical healing and improvement of pain level in children with respiratory pathologies.

A further interpretation of the data must be handled with caution. It is sometimes theorized that if humor does, in fact, decrease stress hormones, this effect is still equivocal, with the few studies conducted demonstrating some conflicting results [15]. Moreover, this is only one mechanism that might explain the proposed connection between humor and improved health outcomes, but not the only one possible, as depicted in Figure 5. 

At the present, it can only be said that humor has a broad range of effects on perceptions, attitudes, judgments and emotions, and these effects can directly or indirectly positively influence the physical and psychological state. The mechanisms underlying these effects are not yet fully understood and additional research is needed in this regard [18].

The present study has some limitations, mainly related to the small sample size, and to the difficulty of carrying out standardized procedures of clown intervention. Moreover, due to a fixed work schedule, a blinding procedure for nurses was not possible in this study. Future research would confirm these results in larger samples and by applying a shared and clearly codified procedure of intervention.

To conclude, our study shows that the presence of clowns in a hospital ward and their interaction with children suffering from respiratory pathologies can improve their clinical recovery. Some positive impact of the clown interaction with the children has been shown at both psychological and physiological level. Humor can be seen as an easy-to-use, inexpensive and natural therapeutic modality to be used within different therapeutic settings, for temporarily alleviating some of the daily distress experienced by different ailing populations.

## Funding

Public funding of “Scuola di Specializzazione di Psicologia della Salute", “Sapienza" Università di Roma to M. B.
